# *Aggregatibacter actinomycetemcomitans* cytolethal distending toxin modulates host phagocytic function

**DOI:** 10.3389/fcimb.2023.1220089

**Published:** 2023-08-31

**Authors:** Taewan J. Kim, Bruce J. Shenker, Andrew S. MacElroy, Samuel Spradlin, Lisa P. Walker, Kathleen Boesze-Battaglia

**Affiliations:** ^1^ Department of Basic and Translational Sciences, School of Dental Medicine, University of Pennsylvania, Philadelphia, PA, United States; ^2^ Department of Periodontics, School of Dental Medicine, University of Pennsylvania, Philadelphia, PA, United States

**Keywords:** phosphoinositide, phagosome maturation, phagocytosis, Cytolethal distending toxin, *Aggregatibacter actinomycetemcomitans*, localized aggressive periodontitis

## Abstract

Cytolethal distending toxins (Cdt) are a family of toxins produced by several human pathogens which infect mucocutaneous tissue and induce inflammatory disease. Human macrophages exposed to *Aggregatibacter actinomycetemcomitans* (*Aa*) Cdt respond through canonical and non-canonical inflammasome activation to stimulate cytokine release. The inflammatory response is dependent on PI3K signaling blockade via the toxin’s phosphatidylinositol-3,4,5-triphosphate (PIP3) phosphatase activity; converting PIP3 to phosphatidylinsoitol-3,4-diphosphate (PI3,4P2) thereby depleting PIP3 pools. Phosphoinositides, also play a critical role in phagosome trafficking, serving as binding domains for effector proteins during phagosome maturation and subsequent fusion with lysosomes. We now demonstrate that *Aa*Cdt manipulates the phosphoinositide (PI) pools of phagosome membranes and alters Rab5 association. Exposure of macrophages to *Aa*Cdt slowed phagosome maturation and decreased phago-lysosome formation, thereby compromising macrophage phagocytic function. Moreover, macrophages exposed to Cdt showed decreased bactericidal capacity leading to increase in *Aggregatibacter actinomycetemcomitans* survival. Thus, Cdt may contribute to increased susceptibility to bacterial infection. These studies uncover an underexplored aspect of Cdt function and provide new insight into the virulence potential of Cdt in mediating the pathogenesis of disease caused by Cdt-producing organisms such as *Aa.*

## Introduction

Periodontitis is a chronic inflammatory disorder mediated by host response to specific oral microbiota (Kornman, 2008; Hienz et al., 2015). In the absence of therapeutic intervention the periodontium (consisting of tooth/teeth, alveolar bone, periodontal ligament and connective tissue attachment) can be damaged, leading to tooth loss (Teughels et al., 2014; Hienz et al., 2015). Stage 3 or 4 and Grade C with a molar-incisor pattern (Tonetti et al., 2018), formerly known as Localized aggressive periodontitis (LAP), is one of the most severe forms of periodontitis. *A. actinomycetemcomitans* (*Aa*), is implicated in the etiology and pathogenesis of LAP as it serves as an early colonizer that facilitates the transition from health to disease. Presumably, *Aa* creates conditions that favor colonization by other organisms; this may include suppression of the host immune system and damage to the periodontium (Fine et al., 2010; Fine et al., 2019; Oscarsson et al., 2019). Among the virulence factors produced by *Aa*, current studies suggest that the cytolethal distending toxin (Cdt) contributes to this altered environment.

Cdt is produced by over 30 γ and ε-Proteobacteria that are human and/or animal pathogens and colonize mucocutaneous tissue such as oral, gastrointestinal, urinary, and respiratory tracts (Johnson and Lior, 1988a; Johnson and Lior, 1988b). Such Cdt-producing bacteria induce disease in these mucocutaneous niches characterized by sustained infection and inflammation. Our recent studies demonstrate that Cdts from *Aa*, *Haemophilus ducreyi* (HdCdt) and *Campylobacter jejuni* (CjCdt) exhibit potent PIP3 phosphatase activity. Moreover, lymphocytes treated with these Cdts exhibit PI-3K signaling blockade: reduced levels of pAkt and pGSK3β (Huang et al., 2021). Previous studies have suggested a novel mechanism of action in which, the active subunit, CdtB, acts as a phosphatidylinositol-3,4,5-triphosphate (PIP3) phosphatase (Shenker et al., 2016). Following internalization, CdtB converts PIP3 to phosphatidylinsoitol-3,4-diphosphate (PI3,4P2) leading to depletions in the PIP3 pools and increases in PI3,4P2. A shift in PIP3 levels not only modulates Akt-GSK3β signaling (Shenker et al., 2014; Boesze-Battaglia et al., 2016; Huang et al., 2021) but also has critical implications for phagocytic function of macrophages that may lead to alterations in phagosome maturation and in turn anti-microbicidal activity (Jeschke et al., 2015; Jeschke and Haas, 2018).

Phosphoinositides (PIs) play an essential role in the trafficking of endosomes and phagosomes by serving as spatio-temporal signposts that direct maturation of phagosomes for content degradation (Jeschke and Haas, 2018; Wallroth and Haucke, 2018). Phagosomes are dynamic structures that interact with endosomes in a process involving acquisition and release of membrane and luminal components as the phagosome matures to a phago-lysosome (Wallroth and Haucke, 2018). This maturation process is controlled by recruitment of proteins, such as Rab5 and Rab7, which are regulated by PI distribution (Fratti et al., 2004; Jeschke and Haas, 2018). Rab5 to Rab7 conversion drives transition of early phagosomes to late phagolysosomes (Fratti et al., 2004; Jeschke and Haas, 2018). PI interconversion can be disrupted either from naturally occurring mutations in PI converting enzymes (phosphatases and kinases) or by experimental manipulation of expression of these enzymes (Vieira et al., 2003; Shin et al., 2005; Wallroth and Haucke, 2018). Such enzymatic defects alter PI distribution, disrupt vesicular transport and are the underlying cause of disease such as oculocerebrorenal syndrome of Lowe, neurological disorders, cancer, and numerous intercellular pathogen related disorders such as tuberculosis, legionellosis, typhoid, and listeriosis (Vicinanza et al., 2008; Yarwood et al., 2020). The alteration in PI pools and the resulting impairment of phago-lysosome formation can significantly adversely affect macrophage host defense function. In this study, we tested that hypothesis that Cdt, via its PI phosphatase activity, hijacks phagocyte maturation thereby creating an intracellular niche that supports *Aa* survival.

## Materials and methods

### Reagents and antibodies

The following antibodies were utilized for western blot and immunofluorescence studies. Mouse anti-PI3,4P2 mAb and mouse anti-PI3,4,5P3 mAb conjugated with FITC from Echelon Bioscience (Salt Lake City, UT). Rabbit anti-EEA1 mAb from Cell Signaling Technology (Danvers, MA). Goat anti-rabbit Ig-HRP conjugate, goat anti-mouse Ig-HRP conjugate, and goat anti-mouse pAb conjugated with Alexa Fluro 488 from Invitrogen (Carlsbad, CA). Rabbit anti-LAMP1 pAb, and rabbit anti-Rab5 pAb from Abcam (Cambridge, England). Mouse anti-Rab7 mAb from Sigma Aldrich (St. Louis, MO). For phagocytosis and phagosome maturation assays, pHrodo™ Red *E. coli* BioParticles™ Conjugate and DQ™-BSA Red (used in 96 well plate assay) and DQ™-BSA -Green (used for live cell assay) was purchased from Invitrogen.

**Table:** 

**Antibody (Host)**	**Source; Catalog #**	**Dilution (application)**
Anti-PI3,4P2 (mouse)	Echelon; Z-P034	1:100 (Immuno)
Anti-PI3,4,5P3 (mouse)	Echelon; Z-G345	1:50 (Immuno)
Anti-EEA1 (rabbit)	Cell Signaling; C45B10	1:100 (Immuno); 1:1,000 (Western)
Anti-LAMP1 (rabbit)	Abcam; ab24170	1:100 (Immuno); 1:1,000 (Western)
Anti-Rab5 (rabbit)	Abcam; ab18211	1:100 (Immuno); 1:1,000 (Western)
Anti-Rab7 (mouse)	Sigma-Aldrich; R8779	1:100 (Immuno); 1:1,000 (Western)

### Cell culture

The human acute monocytic leukemia cell line, THP-1, was obtained from ATCC (Manassas, VA); cells was maintained in RPMI1640-containing 10% FBS, 1 mM sodium pyruvate, 20μM 2-mercaptoethanol and 2% penicillin-streptomycin at 37°C with 5% CO_2_ in a humidified incubator. THP-1 cells were differentiated into macrophages on 35mm glass bottom dishes (MatTek; Ashland, MA), 12 wells cell culture plate (Thermo Scientific; Waltham MA), or 96 wells cell culture plate (Thermo Scientific; Waltham MA) by incubating cells in the presence of 50ng/ml PMA for 48 hours at which time the cells were washed and incubated an additional 24 h in medium prior to use (Shenker et al., 2014).

### Expression and purification of Cdt, CdtB mutants and Cdt holotoxin

Construction and expression of the plasmid containing the cdt genes for the holotoxin (pUCAacdtABChis) have previously been reported (Shenker et al., 2004). The plasmid was constructed so that cdt genes were under control of the lac promoter and transformed into *E. coli* DH5α. Cultures of transformed *E. coli* were grown in 1L LB broth and induced with 0.1 mM of isopropyl β-D-1-thiogalactopyranoside for 2 h; bacterial cells were harvested, washed, and resuspended in 50 mM of Tris (pH 8.0). The cells were frozen overnight, thawed, and sonicated. The histidine-tagged peptide holotoxin was isolated by nickel affinity chromatography as previously described (Shenker et al., 2000).

### Measurement of cellular PI(3,4,5)P3, PI(4,5)P2, and PI(3,4)P2 content

Cells (1×10^6^ cells/well) were incubated with Cdts or media for 240 min. Replicate cultures (0.5–1×10^7^cells) were pooled and harvested. Lipids were extracted as described (Shenker et al., 2014).. The cell pellet was treated with cold 0.5 TCA for 5 min, centrifuged and washed twice with 5% TCA containing 1 mM EDTA. Neutral lipids were extracted twice with methanol:chloroform (2:1) at room temperature. Acidic lipids were extracted with methanol:chloroform:12M HCl (80:40:1) for 15 min at room temperature; the samples were centrifuged for 5 min and the supernatant recovered. The supernatant was then treated with 0.75 ml chloroform and 0.1 M HCl and centrifuged to separate organic and aqueous phases; the organic phase was collected and dried. The dried lipids were resuspended in 120μl 50 mM Hepes buffer (pH 7.4) containing 150 mM NaCl and 1.5% sodium cholate and left overnight at 4°C. PI(4,5)P3, PI(3,4)P3, and PI(3,4,5)P3 levels were then determined using commercially available competitive ELISA according to the manufacturer’s directions (Echelon).

### Confocal microscopy

#### Phosphoinositide immunofluorescence

Cells were treated with Cdts and fixed with 2% PFA and permeabilized with 0.05% saponin. After blocking with 10% goat serum (SouthernBiotech; Birmingham, AL), samples were incubated with mouse anti-PI(3,4)P2 mAb or mouse anti-PI(3,4,5)P3 mAb conjugated with FITC for 1 hour at 37°C (Lally et al., 2020). Tris-buffered saline (TBS) with 1% goat serum was used for washing. For PI(3,4)P2, secondary antibody (goat anti-mouse) conjugated to Alexa Fluor 488 was used while no secondary antibody was used for PI(3,4,5)P3 which was directly conjugated with FITC. For nuclear staining, Hoechst 33258 (AnaSpec Inc; Freemont, CA) was included for all the sets (Boesze-Battaglia et al., 2020). Cells were washed with TBS and imaged within 24 hours.

#### Latex bead – protein association

Cells were treated with Cdts and human serum type AB (Atlanta biologicals S40110; Kolkata, India), opsonized red (ΛEx 580nm/ΛEm 605nm) 1μm diameter latex beads (Invitrogen F13083) were fed to stimulate phagocytosis. At time points indicated in figure legends cells were fixed with 4% PFA and blocked in 0.1% goat serum and 0.1% saponin and incubated with rabbit anti-Rab5, rabbit anti-EEA1, rabbit anti-LAMP1 or mouse anti-Rab7 for 18 hours at 4°C. After primary antibody incubation, cells were stained with Hoechst 33258 (AnaSpec Inc; Freemont, CA) and anti-rabbit or anti-mouse secondary antibody conjugated with Alexa-flour 488 for 1 hour at 37°C, washed with PBS, and imaged within 24 hours (Boesze-Battaglia et al., 2017).

#### Confocal imaging

Images were captured with a Nikon A1R laser scanning confocal microscope with a PLAN APO VC 100×oil (NA 1.45) objective at room temperature. Image z-stacks were acquired at an interval of 0.1μm (50 focal planes/image stack, 5μm). Data were analyzed using Nikon Elements AR 4.30.01 software (Reyes-Reveles et al., 2017). For quantification of phosphoinositide studies, we summed of all focal planes fluorescence intensity and divided by the number of cells in each field. For quantification of latex bead-PI(3,4)P2 association and latex bead–protein association studies, we measured fluorescence intensity around the latex bead (1μm diameter). An area of 4μm^2^ (2x2) around the bead was defined as region of interest (ROI) and summation of fluorescence intensity across the length of the z-stack was used for quantification (Magenau et al., 2011; Segawa et al., 2014; Kissing et al., 2015).

Live cell imaging, THP-1 macrophages were differentiated on 35mm glass bottom dishes and treated with 500ng/ml CdtB^WT^ or media change (untreated control) for 4 hours. pHrodo™ Red *E. coli* BioParticles™ conjugate or DQ™ Green BSA was added for 30min at 4°C and moved to Cytoseal mounting medium (Electron Microscopy Sciences, Hatfield, PA) set at 37°C with 5% CO_2_. Images were captured with a Nikon A1R laser scanning confocal microscope with a PLAN APO VC 20×air (NA 0.75) every 30 minutes and entire plane fluorescence intensity was measured for quantification (Kapellos et al., 2016).

### Western blot analysis

Cells were treated with Cdts and solubilized in 20mM Tris-HCl buffer (pH7.5) containing 150 mM NaCl, 1 mM EDTA, 1% NP-40, 1% sodium deoxycholate, and protease inhibitor cocktail (ThermoFisher Scientific; Waltham, MA). Samples (15μg) were separated on 12% SDS-PAGE and then transferred to PVDF membranes. The membrane was blocked with BLOTTO and then incubated with primary antibodies overnight (18hrs) at 4^°^C (Boesze-Battaglia et al., 2017). Membranes were washed and incubated with secondary antibodies conjugated to horseradish peroxidase (Boesze-Battaglia et al., 2017). Western blots were developed using chemiluminescence and analyzed by digital densitometry (LiCor Biosciences; Lincoln, NE) (Shenker et al., 2020). Each protein was normalized to either actin or GAPDH.

### Assessment of phagocytosis and phagosome maturation activity in THP-1 cells

#### Phagocytosis assay

THP-1 macrophages were differentiated on 96-wells plate. Cells were treated with Cdts with different concentrations as indicated for 4 hours and pHrodo™ Red *E. coli* BioParticles™ Conjugate was applied to measure the phagocytic response. Fluorescence, indication of decrease in pH, was measured at 180 minutes (ΛEx 544nm/ΛEm 590nm) with Multiskan FC (Thermo Scientific) (Kapellos et al., 2016).

#### Phagosome maturation assay

THP-1 macrophages were differentiated on 96-wells plate. Cells were treated with Cdts with different concentrations as indicated for 4 hours and DQ™ Red BSA was applied to measure the phagosome maturation. Fluorescence, indication of phagolysosome formation, was measured at 180 minutes (ΛEx 590nm/ΛEm 620nm) with Multiskan FC (Thermo Scientific) (Frost et al., 2015; Frost et al., 2017).

### Bacteria and growth curve

*A.actinomycetemcomitans* strains, D7S-SA (wild type *Aa*) and D7S-SA CHE001 (Cdt deficient *Aa* mutant), were obtained as described by Nalbant, A., et al (Nalbant et al., 2003). Each strain was plated on AAGM agar which consisted of 20g of BBL trypticase soy agar (BD; Sparks, MD); 3g of yeast extract (ThermoFisher) supplemented with 0.4% sodium bicarbonate and 0.8% dextrose (Sreenivasan et al., 1993). After bacteria were grown on plates for 24 or 48 hours in the incubator with 10% CO_2_ at 37°C, they were inoculated in 10ml of AAGM broth until OD_600_ close to 0.2. Bacteria were plated from different dilutions at various time points on AAGM agar plates and incubated for 24 hours in the incubator with 10% CO2 at 37°C (Nalbant et al., 2003). OD_600_ was measured using DU 650 Spectrophotometer (Beckman Coulter, Indianapolis IN) for each time point.

### Bacterial survival in THP-1 macrophages

THP-1 macrophages (10^6^cells/well) were differentiated on 12 well plates and incubated in the presence of medium only or with Cdt for 240 min. THP-1 macrophages were washed three times with cell media without penicillin/streptomycin prior to inoculation. *Aa* strains were cultured until log phase (OD_600_ of 0.5 to 1) and used to inoculate with Multiplicity of Infection (MOI) (eukaryotic cells: bacteria) of 1:10 for 12 hours at 37°C with 5% CO_2_ in media without penicillin/streptomycin. Cells were then washed with PBS three times and treated with gentamicin (50μg/ml) to wash away and kill extracellular bacteria (Ando-Suguimoto et al., 2014; Sharma and Puhar, 2019). Soy broth with 1% saponin (500μl/well) was added and the mixture incubated at 37°C with 10% CO_2_ for 15 min to lyse macrophages (Perez-Stuardo et al., 2019). Lysates were plated (100μl/plate) on AAGM agar plates and incubated for 1 to 2 days at 37°C with 10% CO_2_. The number of survived bacteria was determined by the number of colonies forming unit (CFU). Same method was applied for 0 hour to measure initial CFU of phagocyted *Aa* population in THP-1 macrophages. Percent of *Aa* surviving after 12 hours was calculated by using equation below:


$$Aa\;12\text{ hours}\;\text{Survival }\left(\%\right)=\;\frac{CFU\;at\;12\;hours\;}{CFU\;at\;0\;hour}*100$$

## Results

### CdtB phosphatase activity increases intracellular membrane PI(3,4)P2 in host macrophages

**Figure 1:**
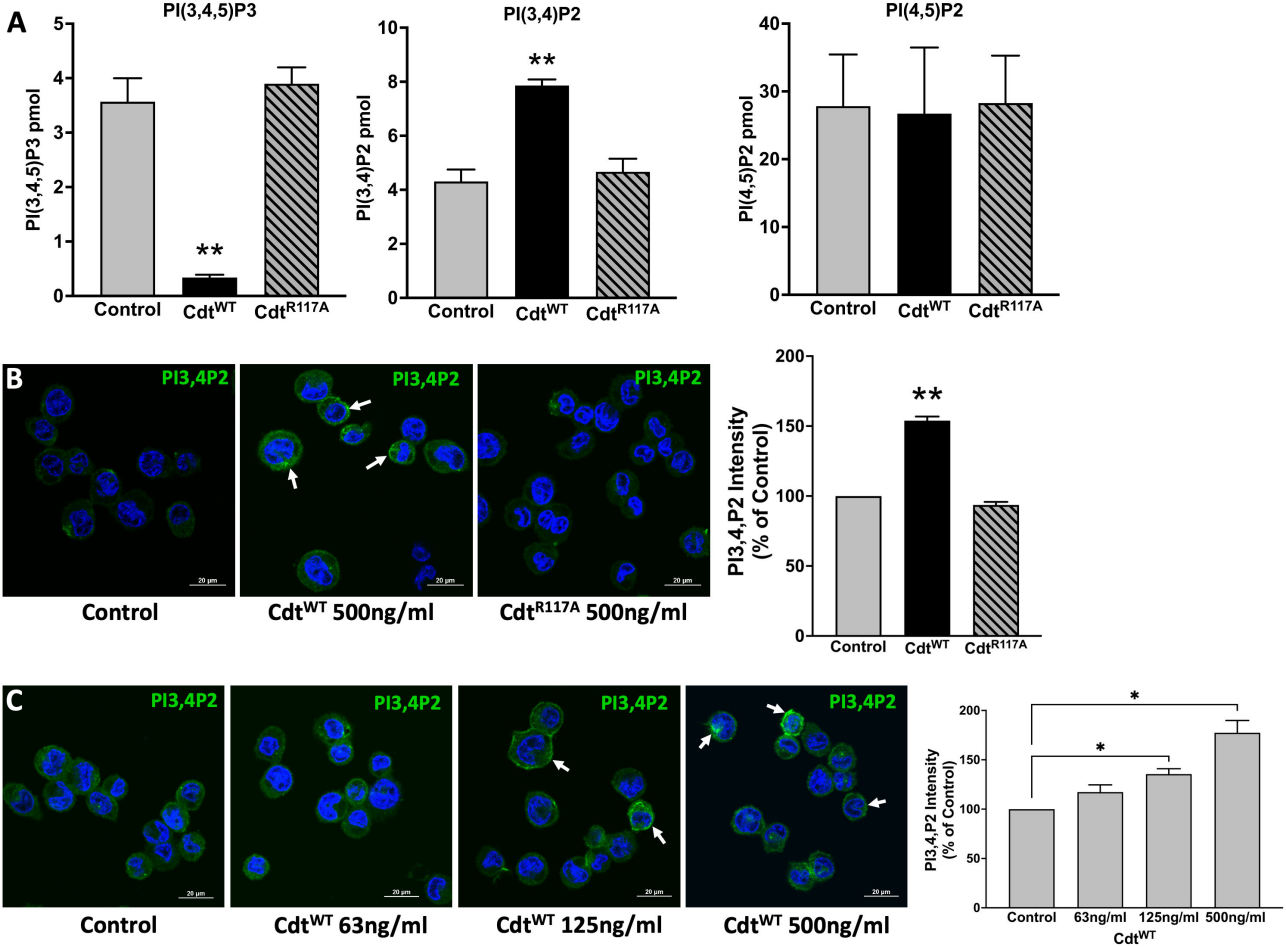
Specificity of *Aa* CdtB phosphatase activity. **(A)** Intracellular PI pools are modulated by CdtB. PI(3,4,5)P3, PI(3,4)P2 and PI(4,5)P2, levels were measured by ELISA in extracts prepared from THP1 macrophages treated with Cdt^WT^ (500 ng/ml) or Cdt^R117A^ (500 ng/ml) as in Methods. Data represent mean +/- STDEV (n=5) and compared using Student’s t-test, **p<0.05. **(B)** Visualization and localization of PI3,4P2 in THP1 macrophages treated with Cdt^WT^ (500 ng/ml) or Cdt^R117A^ (500 ng/ml). PI3,4P2 (Green) as indicated by white arrows, Nucleus (Blue). Quantification of PI(3,4)P2 florescence intensity presented as percent relative to control (untreated). Data represent mean +/- STDEV (five fields with average of 12 cells/field) and compared using Student’s t-test, **p<0.05. **(C)** *Aa* Cdt modulates PI3,4P2 intracellular pools in dose dependent manner. THP1 macrophages were treated with Cdt^WT^ (63ng/ml, 125ng/ml, 500ng/ml) and control (untreated) as in Methods. Visualization of PI3,4P2 level in dose dependent manner, PI3,4P2 (green, as indicated by white arrows), Nucleus (Blue). Quantification of PI(3,4)P2 fluorescence intensity presented as percent relative to the control (untreated). Results are expressed as percent of control (untreated), mean +/- STDEV (five fields with average of 12 cells/field) and compared using Student’s t-test. *p-value<0.05 vs. untreated control.

Cdt treated macrophages exhibit perturbations in intracellular PI pools consistent with the CdtB subunit’s function as a PIP3 phosphatase (Shenker et al., 2014). Cells were exposed to either Cdt holotoxin containing the wildtype CdtB submit (Cdt^WT^) or Cdt holotoxin containing the phosphatase deficient CdtB subunit (Cdt^R117A^). Macrophages treated with Cdt^WT^ exhibited a two-fold increase in PI(3,4)P2 levels with a corresponding decrease in PI(3,4,5)P3 levels (**Figure 1A**). As expected due to CdtB’s enzymatic 5’-phosphatase specificity, no change in PI(4,5)P2 was observed (**Figure 1A**). Furthermore, no change in PI(3,4)P2 or PI(3,4,5)P3 levels was observed in cells treated with Cdt^R117A^ (**Figure 1A**). The increase in PI(3,4)P2 observed with Cdt^WT^ appeared to be associated with intracellular and plasma membrane regions (indicated by white arrows, **Figure 1B**); semi-quantitative analyses indicated a 50% increase in PI(3,4)P2 association within these regions. No increase in PI(3,4)P2 levels were observed in cells treated with CdtB^R117A^. **Figure 1C**, showed distinct PI(3,4)P2 domains (white arrows) with CdtB^WT^ concentrations of 125ng/ml and higher. There was a ~35% increase in PI(3,4)P2 with 125ng/ml and an almost 80% increase when cells were treated with 500ng/ml Cdt^WT^ (Shenker et al., 2016).

### Treatment with Cdt decreases macrophage phagocytic function

**Figure 2:**
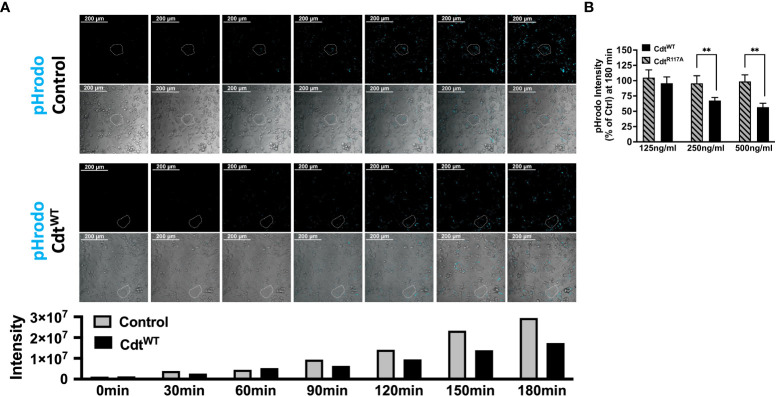
Cdt Phosphatase activity modulates host phagocytic activity. **(A)** Time lapse imaging of pHrodo™ in THP1 macrophages pre-treated with Cdt^WT^ or control (untreated) for 4 hours. pHrodo™ fluorescence (pseudo cyan colored from red) was measured 30 minutes over 3 hours (Top panel: Control (untreated), Bottom panel: Cdt^WT^ treated). Exemplar cells are outlined with white dotted boarder. Intensity measurements are plotted over the experimental time course in the corresponding graph. **(B)** pHrodo™ intensity at 180 minutes, using a 96 well plate format is shown as a function of increasing concentrations of Cdt^WT^ or Cdt^R117A^ as percent of control (untreated) as described in Methods. Data represent mean +/- STDEV (5 wells per individual experiment with 1x10^6^ cells/well) and compared using Student’s t-test. **p-value<0.005 Cdt^WT^ vs Cdt^R117A^.

Having established that Cdt treatment of THP1 macrophages increases intracellular pools of phosphoinositides involved in phagosome processing, we next assessed the ability of Cdt to modulate macrophage phagocytic function with an opsonized pHrodo™ red E. coli BioParticles™ conjugate using live cell imaging. In preliminary experiments we determined that 10μg/ml pHrodo™ was ideal for imaging studies, (data not shown). The red pH-sensitive fluorogenic dye contributing to pHrodo™ intensity (cyan pseudo colored for visualization) was followed for 180min. During this time, pHrodo™ intensity in control (untreated) cells started to dramatically increase at 90 min (4.5x10^6^ to 9.4x10^6^ relative intensity) reaching 2.9x10^7^ relative intensity units by 180 min. In contrast, when cells were pre-treated with Cdt^WT^ (500ng/ml) the magnitude of change in pHrodo fluorescence intensity was comparatively suppressed with minimum of 1.3x10^6^ relative intensity to maximum of 1.7x10^7^ relative intensity, at 180 min. The maximum intensity observed with Cdt^WT^ treatment was 40% less than of that observed in control at 180min (**Figure 2A**), suggesting decreased phagocytic function. In order to conserve materials in our analysis of phosphatase deficient CdtB subunit (Cdt^R117A^), we turned to a 96 well plate format. Macrophages were pre-treated with Cdt^WT^ or Cdt^R117A^, at concentrations of 125-500ng/ml, prior to the addition of pHrodo™. Macrophage phagocytic function, represented by pHrodo™ fluorescence intensity, decreased by over 40% in cells pretreated with 250ng/ml Cdt^WT^ and by over 50% with 500ng/ml Cdt^WT^ (**Figure 2B**). There was no change in pHrodo intensity compared to controls when cells were pretreated with Cdt^R117A^ at any concentration studied (**Figure 2B**). These studies suggest modulation macrophage phagocytic function was dependent on CdtB phosphatase activity.

### Cdt delays steps in phagosome maturation

**Figure 3:**
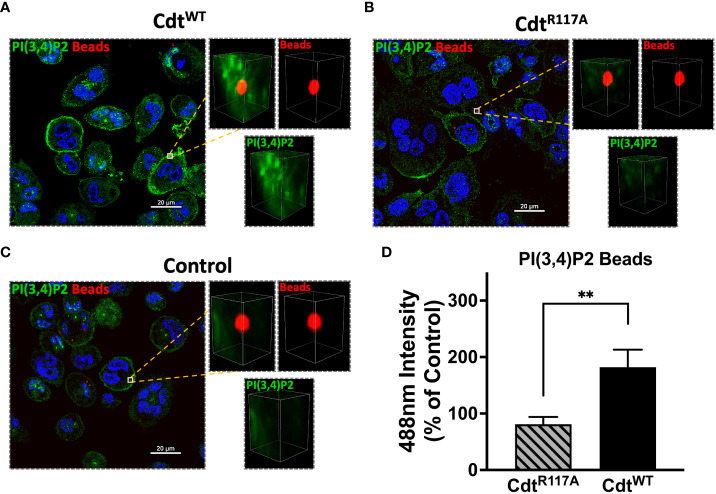
PI(3,4)P2 phagosome association increases with Cdt treatment. THP1 macrophages treated for 4 hours with Cdt^WT^ **(A)**, Cdt^R117A^ **(B)**, or control (untreated, **C**) prior to synchronized uptake of opsonized beads were fixed and stained for PI(3,4)P2 as in Methods. Green: PI(3,4)P2, Blue: nucleus, and Red: opsonized beads. **(D)** Quantified data represent average +/- STDEV of PI(3,4)P2 intensity in 2x2μm area (square) around the bead (10 beads for each for the conditions from 5 random fields) as a percent to control and compared using Student’s t-test. **p<0.05, vs Cdt^R117A^.

The pHrodo™ studies suggested that Cdt^WT^ may modulate phagosome maturation, a process during which effector proteins associate with specific phosphoinositide pools. We sought to determine if there was a spatio-temporal relationship between phosphoinositide pools and phagocytic cargo, specifically ingested latex beads. We first wanted to determine if Cdt treatment altered the composition of phosphoinositides associated with phagosomes. Using opsonized red latex beads (1μm diameter) as the phagocytic cargo, we analyzed the region around the phagocytosed bead for phosphoinositide association using multi-fluor confocal imaging. To assess difference in latex bead-PI(3,4)P2 association in the presence of Cdt^WT^, macrophages were pre-treated with 500ng/ml of Cdt^WT^, Cdt^R117A^, or no treatment (control) for 4 hours. Using a synchronized challenge protocol, opsonized latex beads were added at 4°C with a 30min incubation and phagocytosis intuited with an increase in temperature to 37°C). After 60 minutes of phagocytosis, we analyzed a region centered around the bead in the Z axis with fluorescence intensity measured within a 2μm x 2μm. A PI(3,4)P2 pool was observed associated with the phagocytosed bead in the presence of Cdt^WT^ (**Figure 3A**). There was no detectable PI(3,4)P2-latex bead association when macrophages were pretreated with Cdt^R117A^ or in untreated controls (**Figures 3B, C**). The observation that Cdt^WT^ enhanced PI3,4 P2 levels in phagosome membranes led us to consider if effector protein association with phagosomes was altered in the presence of toxin.

**Figure 4:**
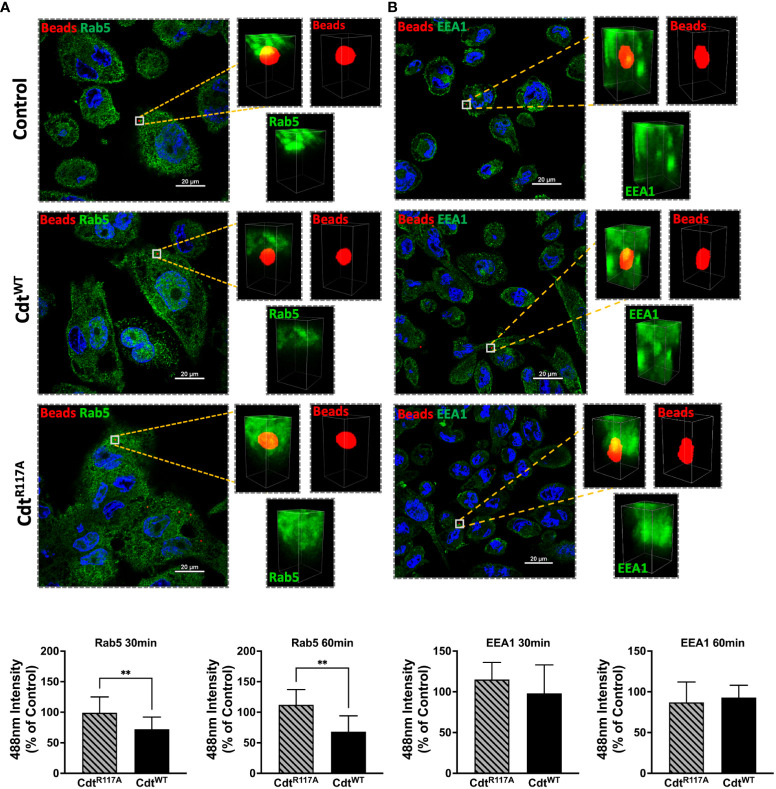
Cdt treatment increases Rab5 phagosome association. THP1 macrophages treated for 4 hours with Cdt^WT^ (500ng/ml), Cdt^R117A^ (500ng/ml) or control (untreated) prior to synchronized uptake of opsonized beads and were fixed and stained for Rab5 and EEA1 as in Methods. Green: Rab5 **(A)** or EEA1 **(B)**, Blue: nucleus, and Red: opsonized beads. Data represent mean +/- STDEV of Rab5 **(A)** or EEA1 **(B)** intensity in 2x2µm area (square) around the bead (10 beads for each for the conditions from 5 different fields) as percent relative to control (untreated) and compared using Student’s t-test. **p-value<0.05 Cdt^WT^ vs Cdt^R117A^.

We next sought to determine if Cdt modulated phagosome association with effector proteins, EEA1, and Rab5. Using opsonized latex beads as the phagocytic cargo, we followed the association of Rab5 and EEA1 with this cargo in the presence of Cdt^WT^ (500ng/ml) using multi-fluor confocal imaging. In these latex bead–protein association studies, we measured fluorescence intensity around the 1μm diameter latex bead. The area of analysis was confined to a region centered around the bead as in the latex bead-PI(3,4)P2 association studies above (**Figure 3**) and described in Methods. In macrophages treated with Cdt^WT^, we observed a decrease in Rab5 association with beads when a 2μm square area around the latex bead was analyzed for Rab5 recruitment. At 30 min, the association of Rab5 with beads decreased by 28% with a further decrease to 32% at 60 min, compared to untreated control cells (**Figure 4A**). Rab5-latex bead association was also unchanged in cells pretreated with Cdt^R117A^ (**Figure 4A**) implicating CdtB phosphatase activity as decreasing dissociation of phagosome-Rab5.

**Figure 5:**
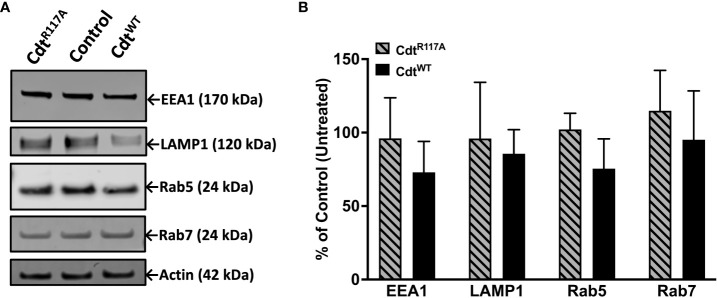
Cdt treatment does not alter levels of intracellular trafficking proteins, EEA1, Rab5, Rab7, and LAMP1. THP-1 macrophages treated with Cdt^WT^, Cdt^R117A^, or control (untreated). **(A)** Cells were challenged with opsonized latex beads for 30 minutes and lysates collected for western blot as in Methods. **(B)** Quantified data represent mean +/- STDEV of EEA1, Rab5, Rab7, and LAMP1 levels (n=3) as percent of control (untreated) and compared using Student’s t-test.

Both Rab5 and EEA1 are associated with phagosomes early in the maturation process. In contrast to Rab5 association, EEA1-latex bead association was unaltered in macrophages treated with Cdt^WT^ or Cdt^R117A^ at both the 30 and 60 min time points (**Figure 4B**). Moreover, no change in latex-bead association with either Rab7 or LAMP1 was observed in Cdt^WT^ or Cdt^R117A^ treated cells (**Supplementary Figure 1**) compared to untreated controls. Neither Cdt^WT^ nor Cdt^R117A^ (500ng/ml) treatment of macrophages altered the levels of any of the intracellular trafficking proteins studied; EEA1, Rab5, Rab7 or LAMP1, compared to untreated control (**Figure 5**). Collectively, these studies suggest that CdtB phosphatase activity dependent changes in phosphoinositide decreases Rab5 association with phagosomes thereby delaying phagosome maturation.

### Treatment with Cdt decreases phago-lysosome formation

**Figure 6:**
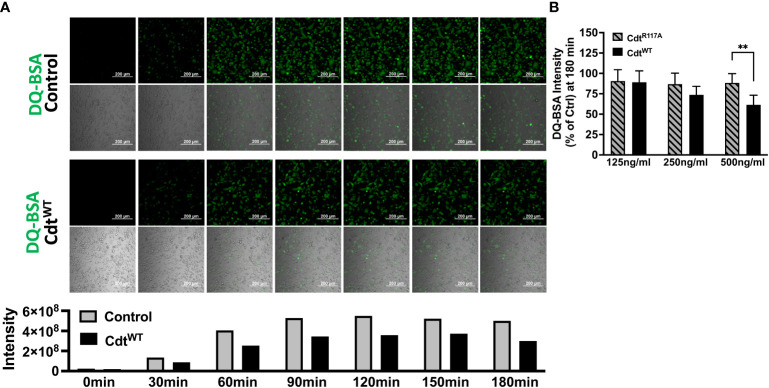
Cdt decreases phagosome maturation. **(A)** Time lapse imaging of DQ™-BSA in THP1 macrophages pre-treated with Cdt^WT^ or control (untreated) for 4 hours. DQ™-BSA fluorescence is indicated by white arrows every 30 minutes over 3 hours (Top panel: Untreated, Bottom panel: Cdt^WT^). Intensity measurements are plotted over the same time course in the corresponding graph. **(B)** DQ™-BSA intensity at 180 minutes, using 96 well plate format is shown as a function of increasing concentrations of Cdt^WT^ or Cdt^R117A^ as percent of control (untreated) as in Methods. Data represent mean +/- STDEV (5 wells per individual experiment with 1x10^6^ cells/well) and compared using Student’s t-test. **p-value<0.005 Cdt^WT^ vs Cdt^R117A^.

The pHrodo™ studies suggested decreased phagocytic capacity but they did not specifically focus on later steps in phagosome maturation. To determine if CdtB phosphatase activity modulates phagolysosome formation, we followed DQ™-BSA fluorescence in macrophages treated with Cdt^WT^ or Cdt^R117A^. Cleavage of the self-quenched DQ™-BSA protease substrate in an acidic compartment generates a highly fluorescent product (Frost et al., 2017). DQ™-BSA intensity was followed for 180min, the control (untreated) cells reached maximum intensity (5.5x10^8^ intensity) by 120min while cells pre-treated with Cdt^WT^ (500ng/ml) reached maximum intensity (3.7x10^8^ intensity) at 150min. Also, when we employed the 96-well plate assay at 180min, macrophages pretreated with Cdt^WT^ (500ng/ml) exhibited a 40% decrease in DQ™-BSA fluorescence (**Figure 6A**) suggesting a reduction in phagolysosome formation. Cdt^R117A^ treatment did not alter DQ™-BSA fluorescence implicating phosphatase activity as the major contributor to decreased phagolysosome formation (**Figure 6B**). Lastly, neither Cdt^WT^ nor Cdt^R117A^, altered lysosome integrity (**Supplementary Figure 2**)

### Effect of Cdt on survival of *Aggregatibacter actinomycetemcomitans* in macrophages

**Figure 7:**
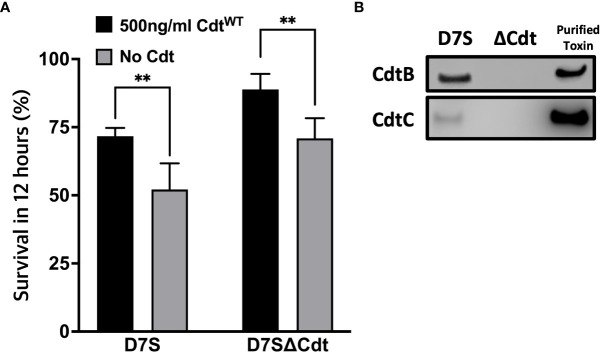
Cdt pretreatment enhances *Aa* survival. **(A)** THP1 macrophages were pre-treated with 500ng/ml Cdt^WT^ for 4 hours or control (untreated). Cells were subsequently incubated with D7S and D7SΔCdt for 0 and 12hours. Cells treated as in Methods and lysates were plated on agar plate and CFU units were determined. Data represent mean +/- STDEV (n=3) as percent of survived within 12 hours as described in Methods and compared using Student’s t-test **p<0.05 vs No Cdt. **(B)** Confirmation of Cdt production in D7S and loss of Cdt in D7SΔCdt strains. D7S and D7SΔCdt were plated on agar and then cultured by broth media. Western blot analysis was performed on bacterial lysates and positive control (purified Cdt) using mouse antibodies for Cdt subunits to confirm the mutations.

The studies detailed above suggest that Cdt^WT^ decreases phagosome maturation by modulating PI pools and decreasing phago-lysosome fusion. In this final series of experiments, we assessed *Aa* survival to determine if Cdt^WT^ alters macrophage bactericidal function. When THP1 macrophages were inoculated with wild type *Aa* (D7S), or the mutant *Aa* lacking Cdt (D7SΔCdt) at a multiplicity of infection (MOI) of 1:10 for 12 hours, both bacterial strains exhibited virtually the same extent of survival when to compared 0 hour (time-zero) CFUs (**Figure 7A**). To determine if macrophage exposure to Cdt, as may occur *in vivo* alters *Aa* phagocytic processing, macrophages were pretreated with Cdt at a concentration shown to decrease phagosome maturation and phago-lysosome fusion (**Figure 6A**). When macrophages were pretreated with Cdt^WT^ (500ng/ml) and subsequently inoculated with either wild type *Aa* or Cdt deficient mutant *Aa*, there was a 17.2% increase in *Aa* (D7S), and an 18.8% increase in *Aa* (D7SΔCdt) survival. Thus, pretreatment of THP1 derived macrophages with Cdt^WT^ resulted in an increase in *Aa* survival and a decrease in bactericidal effect (**Figure 7A**). When we utilized an MOI of 1:100 for 12 hours, macrophage cell death was observed (data not shown).

## Discussion

In the oral cavity, *Aa* is a risk factor for gingival inflammation and localized aggressive periodontitis (Stage 3 or 4 and Grade C with molar-incisor pattern); we propose that Cdt contributes to the virulence properties of *Aa* (Tonetti et al., 2018; Fine et al., 2019). We have documented a novel intoxication profile for Cdt, as a tri-perditious toxin and shown that the manner of intoxication is cell type specific (Shenker et al., 2014; Scuron et al., 2016). Human monocytes and macrophages are resistant to *Aa-*Cdt induced apoptosis, while lymphocytes become apoptotic (Shenker et al., 2005; Scuron et al., 2016). In macrophages, we show Cdt, induces a robust pro-inflammatory response that involves activation of the NLRP3 inflammasome as well as the non-canonical inflammasome with cytokine release dependent on gasdermin cleavage (Shenker et al., 2020).

**Figure 8:**
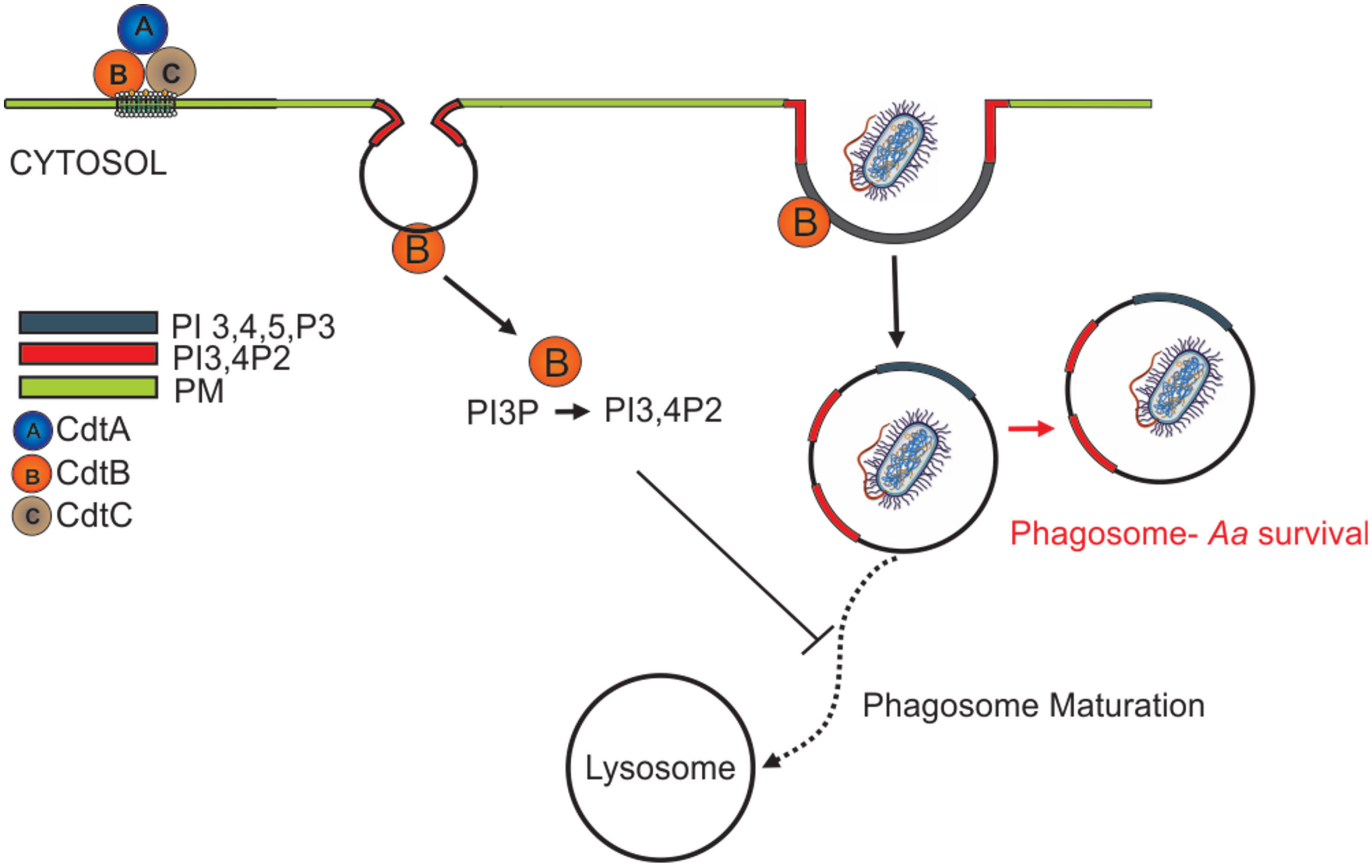
Overview of Cdt internalization and the effect on the phagosome. After the internalization, CdtB act as a phosphatase and convert the PI(3,4,5)P3 to PI(3,4)P2. Change in the PI pool leads to manipulation of phagosome maturation.

Ando-Suguimoto, and colleagues suggested that the Cdt may modulate macrophage function in *Aa* infected sites by impairing phagocytosis using murine macrophages (Ando-Suguimoto et al., 2014). Our current studies expand on this observation to describe the molecular mechanism by which Cdt exerts its effect and contributes to *Aa* pathogenicity by creating an intracellular survival niche in human macrophages. We have shown that once internalized, the active CdtB subunit alters intracellular phosphoinositide pools, with the enzymatic depletion of PIP3 resulting in blockade of the PI-3K signaling pathway leading to both Akt inactivation and GSK3β activation (Shenker et al., 2016). In these studies, we explored the effect of Cdt mediated changes in phosphoinositide pools on the modulation of host phagocytic function. Intracellular phosphoinositide pools not only serve as signaling platforms but also mediate phagosome maturation and pathogen degradation (Jeschke et al., 2015; Jeschke and Haas, 2018; Wallroth and Haucke, 2018). Phagosomes are dynamic structures that interact with endosomes in a process involving acquisition and release of membrane and luminal components as the phagosome matures to a phago-lysosome (Jeschke et al., 2015; Jeschke and Haas, 2018; Wallroth and Haucke, 2018). This maturation process is controlled by recruitment of proteins, such as Rab5 and Rab7 (and other effector proteins), which is regulated by PI distribution (Vieira et al., 2003; Shin et al., 2005). Specific phosphoinositide species act as zip-codes ensuring that the maturing phagosome is targeted towards lysosomes for fusion and degradation as illustrated schematically in **Figure 8**. PI(3,4,5)P3 and PI(4,5)P2 play critical roles in phagosome formation and PI3P and PI(3,4)P2 in phagosome maturation.

Herein we show that Cdt^WT^, as expected stimulates a decrease in PI(3,4,5)P3 and a corresponding increase in PI(3,4)P2 which is localized to distinct domains and surrounds phagocytosed latex beads (**Figures 1**, **3**). The extent of PI(3,4)P2 association with latex beads is clearly visible in the presence of Cdt^WT^ but is undetectable in cells treated with Cdt^R117A^ or in untreated cells (**Figure 3**). Importantly, PI(4,5)P2 levels remained unaltered (**Figure 1**) and pHrodo™ (**Figure 2**) and DQ™-BSA (**Figure 6**) uptake did not vary between Cdt^WT^ and Cdt^R117A^ suggesting that the initial step in uptake, a PI4,5,P2 dependent process that recruits actin-modifying proteins is intact. Our Rab5 and EEA1 latex bead association studies suggest that Cdt^WT^ phosphatase activity modulates a step downstream of uptake in the maturation process. EEA1 recruitment to latex beads was not sensitive to Cdt^WT^ treatment (**Figure 5B**). In contrast association of Rab5, an early phagosome/endosome associated GTPase with latex beads decreased upon Cdt^WT^ treatment Rab5-latex bead association was decreased (**Figure 5A**). Whether this decrease is due to alteration in the levels of PI3P, is currently under investigation. Early Rab5 positive endosomes/phagosomes subsequently become late Rab7 positive structures (Vieira et al., 2003; Mottola, 2014). Thus, diminished Rab5 phagosome association can be indicative of stalled phagosome or slowed phagosome maturation. In fact, Cdt^WT^ treatment resulted in decreased phagosome maturation and fusion with lysosomes as indicated in **Figure 6**. Collectively, these studies provide a molecular mechanism to explain diminished phagocytic capacity observed in murine macrophages upon *Aa*Cdt treatment (Ando-Suguimoto et al., 2014).

These studies provide molecular insight into how *Aa*Cdt subverts phagocytosis and creates a survival niche for subsequent infections (**Figure 7**). *Aa*, specifically due to its CdtB phosphatase activity, can be added to the rapidly growing list of pathogens that have evolved to evade antimicrobial effects of macrophages thus crippling the effective immune response (Sarantis and Grinstein, 2012). In the context of periodontal disease, this effect of *Aa*Cdt provides a molecular mechanism by which *Aa* functions in the oral cavity to provide a “survival” niche in the gingival crevice for other pathogenic microorganisms which collectively contribute to LAP pathogenesis (Loe and Brown, 1991; Teughels et al., 2014; Fine et al., 2019). *Aa* is present in healthy oral flora, however the transition from health to disease is reflected in the habitat within which *Aa* exists (Fine et al., 2019). Under healthy conditions, *Aa* dwell and colonize above the gum-line. In the disease state, *Aa* transits below the gum-line, to the subgingival niche, an area with less oxygen (Fine et al., 2019). This localization favors *Aa* as it is a facultative anaerobic gram-negative bacterium. *Aa* utilize three major toxins. apiA gives adhesion, invasion, and resistance to the complement system (Fine et al., 2019). Leukotoxin causes leukocyte apoptosis (Korostoff et al., 1998; Oscarsson et al., 2019). Cdt causes cell cycle arrest, apoptosis in T cells and epithelial cells, upregulates the pro-inflammatory cytokines in macrophages (Shenker et al., 2005; Shenker et al., 2014; Scuron et al., 2016), and with current study, it also decreased the phagocytic effect of macrophage. The new finding can suggest an idea that *Aa*Cdt and leukotoxin can work synergistically as leukotoxin causes endocytic vesicles and lysosomal rupture at a low pH thus further impairing the phagocytic degradation (Lally et al., 2020). We predict that these disruptions could impair the body’s defense system by decreasing the antimicrobial effect, causes dysbiosis, and exacerbate the inflammatory response. These combined effects of *Aa* strongly suggest that it may be an initiator of LAP thereby preparing the niche for other pathogens. This interpretation can also be applicable to other Cdt producing pathogen as a new mechanism of hijacking host function to create pathologically favorable condition.

Lastly, Our observation that CdtB phosphatase activity slows phagosome maturation likely through a Rab5 effector provides a molecular mechanism for the observation that *Campylobacter jejuni*, Cdt show elevated toxicity in cells over expressing Rab5 (Chen et al., 2020). We predict this is due to CdtB-mediated decreased phagosome maturation hence increased *Campylobacter jejuni* survival and this prediction remains to be tested.

## Data availability statement

The raw data supporting the conclusions of this article will be made available by the authors, without undue reservation.

## Author contributions

TJK, KB-B, and BJS conceptualized the study. KB-B and BJS provided oversight and funding acquisition for the study. TJK, LPW, and ASM contributed to the methodology. TJK, ASM, SS and LPW contributed to data and figure curation. TJK and KB-B wrote the original draft. TJK, KB-B, and BJS contributed to the writing, review and editing of the manuscript. All authors contributed to the article and approved the submitted version.

## Supplementary material

Supplementary Figure 1 Effector proteins, Rab5, EEA1, and Rab7 levels were unaltered upon Cdt treatment. THP1 macrophages treated for 4 hours with Cdt^WT^, Cdt^R117A^, or control (untreated) prior to synchronized uptake of opsonized beads were fixed (at 30min and 60min for Rab5 and EEA1, at 1hr and 3hr for Rab7) and stained for Rab5, EEA1, and Rab7 as in Methods. Data represent mean +/- STDEV of protein intensity (5 different fields with about 12 cells/field) as percent to control and compared using Student’s t-test.

Supplementary Figure 2 Cdt treatment does not alter LAMP1. **(A)**. LAMP1 staining in THP1 macrophages pre-treated with Cdt^WT^ (500ng/ml), Cdt^R117A^ or control (untreated) prior to the addition of opsonized beads as in Methods. **(B)**. LAMP1 fluorescence intensity at 1 hour **(B)** or 3 hours **(C)**. Data represent mean +/- STDEV of LAMP1 intensity (5 fields per condition with average of 12 cells/field) as a percent to control and compared by using Student’s t-test.
