# Corrigendum: *Aggregatibacter actinomycetemcomitans* cytolethal distending toxin modulates host phagocytic function

**DOI:** 10.3389/fcimb.2023.1321218

**Published:** 2023-10-25

**Authors:** Taewan J. Kim, Bruce J. Shenker, Andrew S. MacElroy, Samuel Spradlin, Lisa P. Walker, Kathleen Boesze-Battaglia

**Affiliations:** ^1^ Department of Basic and Translational Sciences, School of Dental Medicine, University of Pennsylvania, Philadelphia, PA, United States; ^2^ Department of Periodontics, School of Dental Medicine, University of Pennsylvania, Philadelphia, PA, United States

**Keywords:** phosphoinositide, phagosome maturation, phagocytosis, Cytolethal distending toxin, *Aggregatibacter actinomycetemcomitans*, localized aggressive periodontitis

In the published article, an author name was incorrectly written as Andrew S. MacElory. The correct spelling is Andrew S. MacElroy.

The authors apologize for this error and state that this does not change the scientific conclusions of the article in any way.

