# Supplementary figures and images for: Aggregatibacter actinomycetemcomitans cytolethal distending toxin modulates host phagocytic function
Source: Front Cell Infect Microbiol. 2023 Aug 31;13:1220089. doi: 10.3389/fcimb.2023.1220089 (PMC10500838; doi:10.3389/fcimb.2023.1220089)

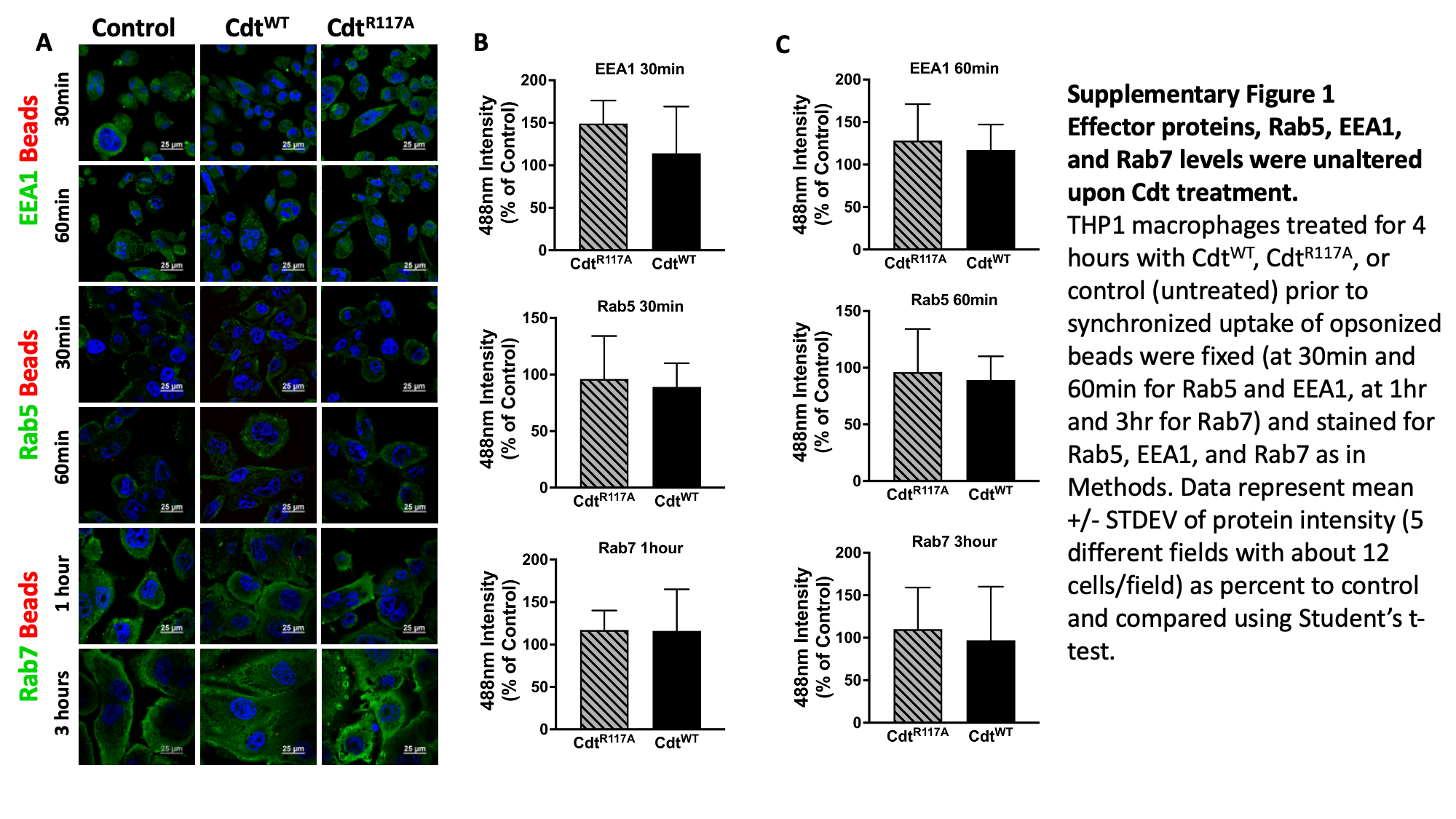

Supplement: Supplementary Figure 1 — Effector proteins, Rab5, EEA1, and Rab7 levels were unaltered upon Cdt treatment. THP1 macrophages treated for 4 hours with CdtWT, CdtR117A, or control (untreated) prior to synchronized uptake of opsonized beads were fixed (at 30min and 60min for Rab5 and EEA1, at 1hr and 3hr for Rab7) and stained for Rab5, EEA1, and Rab7 as in Methods. Data represent mean +/- STDEV of protein intensity (5 different fields with about 12 cells/field) as percent to control and compared using Student’s t-test. [file Image_1.tiff]

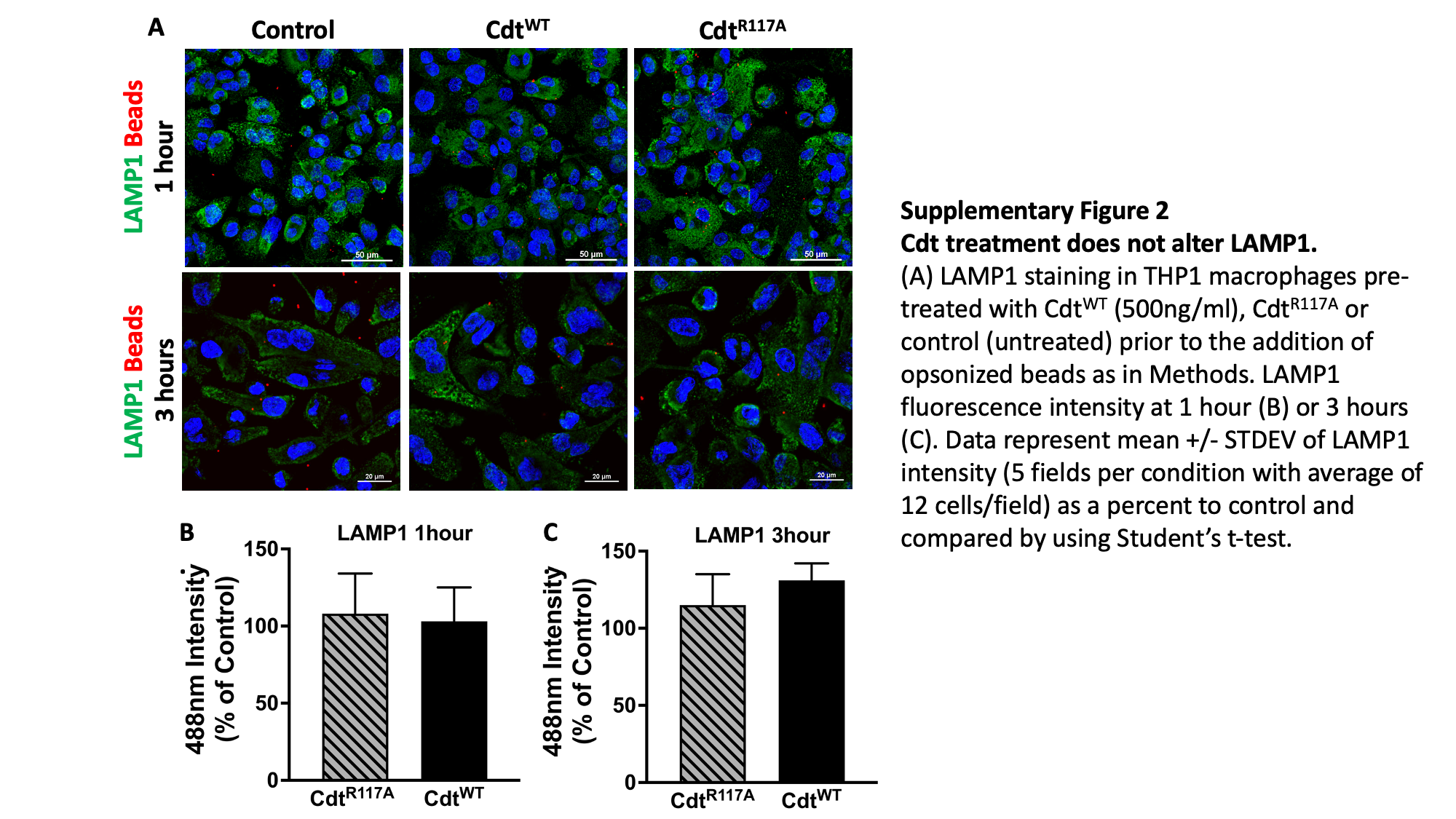

Supplement: Supplementary Figure 2 — Cdt treatment does not alter LAMP1. (A). LAMP1 staining in THP1 macrophages pre-treated with CdtWT (500ng/ml), CdtR117A or control (untreated) prior to the addition of opsonized beads as in Methods. (B). LAMP1 fluorescence intensity at 1 hour (B) or 3 hours (C). Data represent mean +/- STDEV of LAMP1 intensity (5 fields per condition with average of 12 cells/field) as a percent to control and compared by using Student’s t-test. [file Image_2.tiff]
